# VCF-Miner: GUI-based application for mining variants and annotations stored in VCF files

**DOI:** 10.1093/bib/bbv051

**Published:** 2015-07-25

**Authors:** Steven N. Hart, Patrick Duffy, Daniel J. Quest, Asif Hossain, Mike A Meiners, Jean-Pierre Kocher

**Keywords:** bioinformatics, genomics, analysis, software, user interface, VCF

## Abstract

Next-generation sequencing platforms are widely used to discover variants associated with disease. The processing of sequencing data involves read alignment, variant calling, variant annotation and variant filtering. The standard file format to hold variant calls is the variant call format (VCF) file. According to the format specifications, any arbitrary annotation can be added to the VCF file for downstream processing. However, most downstream analysis programs disregard annotations already present in the VCF and re-annotate variants using the annotation provided by that particular program. This precludes investigators who have collected information on variants from literature or other sources from including these annotations in the filtering and mining of variants. We have developed VCF-Miner, a graphical user interface-based stand-alone tool, to mine variants and annotation stored in the VCF. Powered by a MongoDB database engine, VCF-Miner enables the stepwise trimming of non-relevant variants. The grouping feature implemented in VCF-Miner can be used to identify somatic variants by contrasting variants in tumor and in normal samples or to identify recessive/dominant variants in family studies. It is not limited to human data, but can also be extended to include non-diploid organisms. It also supports copy number or any other variant type supported by the VCF specification. VCF-Miner can be used on a personal computer or large institutional servers and is freely available for download from http://bioinformaticstools.mayo.edu/research/vcf-miner/.

## Introduction

Next-generation sequencing (NGS) is widely used to study associations between genetic variation and diseases. When these NGS platforms became available, the first concern was related to the amount of generated data requiring management and processing. Most academic institutions have addressed this issue by expanding the information technology infrastructure with computing and storage resources. A growing number of optimized applications and workflows, designed for the effective alignment of sequence reads and calling of variants [1, 2], have also become available.

The focus has now shifted to the interpretation of the called variants. The first step consists of annotating and filtering variants in an attempt to weed out those with low relevance to the disease in question. Several tools, including BioR [3], ANNOVAR [4] and variant call format (VCF) tools [5], consolidate annotations from multiple sources and have been designed to simplify the annotation task. These tools derive annotations from the analysis of the aligned reads (e.g. variant quality score) or the predicted impact of missense mutations (Condel [6], PolyPhen2 [7] and EvoD [8]). They also consolidate annotation from public data sources (Exome Sequencing Project [9], the 1000 Genomes Project [10] and the Exome Aggregation Consortium [11]) and can integrate private annotations such as institution-specific rare variant information or variants associated with institution-specific sequencing artifacts. To ensure the integrity of the association between variants and annotations, most of these annotation tools leverage the flexibility of the VCF file to store variants and related annotations. This additional level of integrity enabled by VCF can be of particular interest in the context of clinical testing because it keeps information on a patient in a single file.

Once annotated, variants should be filtered to eliminate those of low relevance. The filtering process remains mostly under the purview of bioinformaticians, as most of the available tools, including SNPsift [12], GEMINI [13] and VCF tools [5], are command line-based, which presents a barrier to investigators. To our knowledge, there are only a few applications available to non-bioinformatician investigators to perform this task. This includes the Ingenuity^®^ Variant Analysis^™^ software [14] from Ingenuity Systems, Golden Helix SNP & Variation Suite [15] and the recently published BiERapp [16]. Although these applications aim to enhance the role of the subject matter expert in the process of identifying relevant variants, they all share a common drawback. They restrict the user to use the annotations they provide. They disregard the annotations embedded in the VCF. This presents a significant limitation, especially when considering the growing interest in incorporating private or user-collected annotation in the filtering process.

In this manuscript, we are presenting VCF-Miner, an open source, Web interface application designed to filter variants based on the annotations included in the VCF file. VCF-Miner includes a powerful filtering engine and sample-grouping feature that can be used to identify somatic variants or recessive/dominant variants in family studies. VCF-Miner keeps track of the filtering history to facilitate retrospective review of the processing steps. The application can filter millions of variants in seconds on a personal computer but can also be deployed on large-scale institutional servers.

## Method

### Loading and processing a VCF

The VCF [5] has become the standard format to store results from small to large genomics projects [10, 17]. Designed to be flexible while compact, the VCF file stores variant-, gene-, sample- and study-related annotations. The first eight columns (a.k.a fields) describe the chromosome (CHROM), starting position of the variant (POS), external identifier tags (ID), the reference position in the genome (REF), what the variant is (ALT), quality metrics (QUAL), whether the variant passes quality control checks (FILTER) and variant-specific annotation (INFO) which can be in key-value pairs or flags (present/absent). Typically, the INFO field is used by annotation tools to store vectors of annotations. The eighth column acts as the key to values from sample-specific data, which begin in Column 9. There is no limit to the number of samples or annotations that can be contained within the VCF file.

VCF-Miner can handle uncompressed or compressed (*.gz) VCF files. During the loaded process, VCF-Miner evaluates metadata in the header of the VCF (or gVCF) to determine the data to extract from the SAMPLE and INFO fields, which are in turn transformed into JSON array of values that are stored and indexed in a MongoDB database. The MongoDB system was chosen for the flexibility and rapid querying and filtering, especially on sparsely annotated data sets typically found in VCF files. Once a VCF is loaded, all of the properly formatted data from the INFO field is available for querying.

For the SAMPLE fields, three types of logic are applied. The first logic is used to parse the genotype field and determine whether the variant is detected in the sample. Specifically, genotype data from the GT field of VCF files are stripped for characters such as ‘.’, ‘/’, ‘|’ and 0, which are not informative of a mutated genotype. If any characters remain, then the number of remaining characters is equal to the number of alternate alleles in the sample. If the number of alternate alleles is >1, the sample is treated as homozygous, and if the number of alternate alleles is equal to 1, then it is heterozygous. The benefit of this logic is that VCF-Miner can handle diploid (0/1) and non-diploid (0/0/1) genotypes. The second type of logic is used to calculate minimum and maximum values for numerical fields from each of the SAMPLE columns. As an example, SoftSearch [18] is a tool that outputs structural variations in VCF format, including the number of reads supporting the event. Once loaded into VCF-Miner, the user can remove structural variants from the SoftSearch VCF that have too few supporting reads in one or more samples. The third logic targets the alternate allele depth field (AD field in the FORMAT columns). VCF-Miner will parse out the number of alleles supporting a variant, assuming that the AD fields include the number of reads supporting the reference allele, followed by the number of reads supporting the alternate allele separated by a comma. These two values are exposed to the user in the user interface, allowing filtering based on alternate allele depth. In terms of best practices, we recommend that annotations are added only to VCF files that contain one allele per row. Simple Perl scripts are sufficient to convert a multi-allelic VCF into a single allele VCF while maintaining the VCF specification. This script is available on request.

### Graphical user interface design

**Figure 1.**
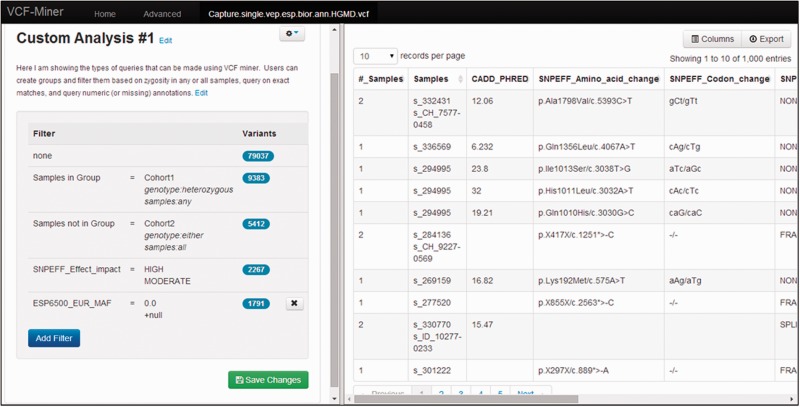
Screenshot of VCF-Miner. The left panel shows a running tabulation of filters applied and the number of variants remaining. A pop-up dialog appears when the user clicks the ‘Add Filter’ button. The right panel consists of a tabular representation of the results. Users can choose which columns to show and hide, and when ready, a tab-delimited file of the selected filtered data and annotations can be exported.

VCF-Miner includes a dashboard-based graphical user interface (GUI) to let non-bioinformaticians query and filter their data (Figure 1). Variants and annotations included in the VCF file are displayed in a tabular form in the right panel of the dashboard. The first set of fields show variant-related annotations. The last two fields display the total number of samples and list of comma-separated sample IDs. Each field can be hidden (or displayed) to let the user focus on the annotation of interest. Each field can also be sorted.

The left panel of the user interface displays the list of filters applied to the data. Filtering can be applied to either variant- or sample-specific annotation fields. Each filter is applied to a single field selected from a scroll down list. Note that hidden fields are included in this list and therefore can be selected, although not visible in the main display. The type of field (i.e. numeric, binary or string) is automatically detected to adjust the set of applicable operators. Numeric fields are filtered using relational operators such as ‘<’, ‘≤’ and ‘=’. Binary fields are filtered based on their ‘True’ or ‘False’ status. For fields containing strings, the interface switches between a drop-down check box (if the number of unique strings selected from is ≤25) and a type-ahead box (when this number is >25). Filters are consecutively applied to the data such that each filter is applied to the variants that have passed previous filter(s). The number of remaining variants is displayed next to each filter. The list of filters can be saved to be reused with this or other projects/VCF files.

**Figure 2.**
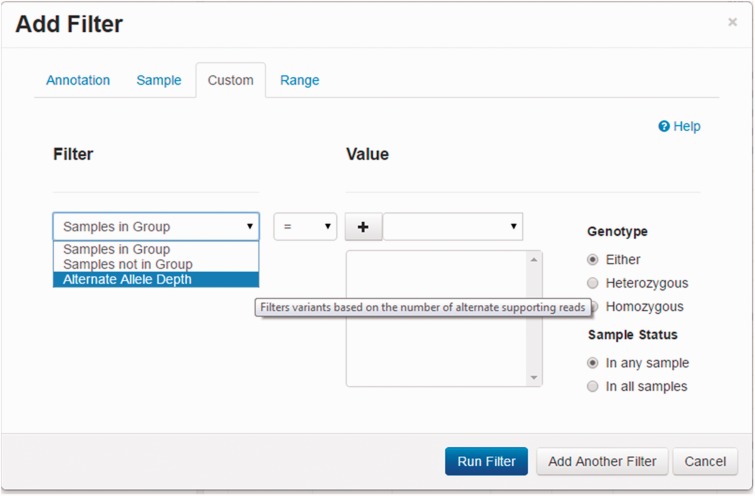
Custom logic filtering. In this figure, we demonstrate how to construct filters across groups of samples. Group 1 consists of nine samples. One could restrict variants to those present in Group 1 using the default setup. By changing the genotype option to heterozygous, then the variants returned would have to be heterozygous in any sample. To return only variants that are heterozygous in all nine samples, the sample status would be changed to ‘In all samples’. The alternate allele depth filter allows the user to specify the minimum number of reads supporting a variant—provided the VCF contains an AD field (see text for more details).

By default, filters are applied to all samples, but users can also create groups of samples. This feature is implemented to support case/control studies where variants present in cases, but absent from controls need to be identified. This feature can also be used to analyze family trios based on a mode of inheritance (Figure 2). For instance, when looking for an autosomal recessive variant, a first group can be created for the parents and another one for the affected offspring. Variants can then be successively filtered requiring first to be heterozygous in both parents followed by homozygous in the child. Users are not restricted to the number of groups that can be created, so more complex groups can be defined and queried—extending the flexibility to more complex biological applications.

Using a case-control scenario, the users could find variants that are homozygous in all cases. They could then filter more variants by excluding those observed in any of the controls. Alternatively, the users could only exclude variants that were homozygous in any of the controls. This flexibility of filtering genotypes on the basis of groups allows for these types of complex queries to be made.

Another feature lets users filter based on genomic ranges. Users can upload BED files what are then converted into annotations (https://genome.ucsc.edu/FAQ/FAQformat.html#format1). Each variant will be labeled as whether the variant belongs (TRUE) or does not belong (FALSE) to the genomics regions listed in the BED file. Users can load as many BED files as necessary, but must give each a distinct name. If only a few genomic ranges are of interest, the user can simply paste in the ranges in the appropriate filter box, without needing to upload a BED file.

Note that Groups as well as the filtering strategy can be saved for later use, and filtering strategies can be exported to be applied to other data sets.

## Results

### Benchmarks

Benchmarks for VCF-Miner’s files of different types are presented in Table 1. The amount of time required to upload a file is dependent on several factors such as the number of samples, annotations and variants. In our tests (files available online), we evaluated several combinations of numbers of variants (10–350 K), annotations from the format and info fields (19–124) and number of samples (1–629). The number of samples in a VCF file is the principle driver of load time. We achieved a loading rate of ∼200 variants/s when the number of samples was 629, but the rate was 1100–2500 variants/s (5–10× faster) when the VCF file contained one sample. Once loaded, queries typically happen in milliseconds, allowing users to rapidly delve into their data interactively. On PC1, we queried variants in the ‘1KG.chr22.anno.infocol.vcf.gz’ file, with and without indexes. Queries were identical—restricting the variants to those with a SAVANT_IMPACT value of HIGH. The non-indexed query took 2.466 ms. After applying the index in the ‘Advanced’ page, we reapplied the SAVANT_IMPACT query, which took 0.106 ms.

**Table 1.** Benchmark results for loading three different VCF files into VCF-Miner

File	Size (MB)	Variants	Format and info fields	Samples	Load time (min)	
					PC1	PC2
Genome In a Bottle.vcf.gz	398	3 315 166	84	1	147	105
1KG.chr22.anno.infocol.vcf.gz	980	348 110	124	629	32.5	42.4
1KG.chr22.anno.vcf.gz	918	346 660	19	629	29	39.7
HG00098.anno.vcf.gz	1.9	46 311	110	1	0.7	0.6
HG00098.vcf.gz	1.2	46 065	23	1	0.3	0.3
1KG.chr22.anno.20kLines.vcf.gz	57	19 876	124	629	1.8	2.5
1KG.chr22.anno.10kLines.vcf.gz	28	9981	19	629	0.8	1.4
1KG.chr22.anno.infocol.10kLines.vcf.gz	29	9876	124	629	0.9	1.1

*Note.* PC1 is an Ubuntu Linux v12.04, AMD64 CPU at 1400 MHz and 96 GB RAM.

PC2 is a laptop running Windows 7 Professional, with an Intel Core i5-4300 CPU at 1.90 GHz and 8 GB RAM.

### Caveats

It should be noted that VCF-Miner is sensitive to the accurate formatting of the VCF. The file must pass validation testing from either the vcf-validate routine from VCF tools (version 1.12a+) [5] or ValidateVariants routine from GATK (version 3.1+) [2], before being loaded into VCF-Miner. If an error in file format arises, the error and offending line are presented to the user. This process requires information lines describing the INFO, FILTER and FORMAT of entries used in the body of the VCF file to be included in the header section. VCF-Miner does not remove the need for a bioinformatician because adding annotations to a VCF are primarily done using command-line tools. However, it does allow the investigator full access to their data, when and where they are ready to analyze it.

## Use cases and practical guidance

To demonstrate the utility of customized annotation, we present two use cases: a trio-based analysis and a large multi-group cohort. At Mayo Clinic, these two analyses are run with different genomics workflows, both producing a VCF. Many annotations in the VCF are the same (e.g. variant frequency in the 1000 Genomes Project, stop gains and PolyPhen2), but several other sets of annotations are added depending on the biological question. These features are added to the VCF file through the BioR toolkit, individual applications or via custom scripts.

In the first scenario, we assume a trio consisting of a mother, father and affected child that has been sequenced. While some software allow genotype-level querying (e.g. heterozygous variant in mother and father while homozygous in child), assessing the relevant combinations of genotypes to the phenotype can quickly become onerous. For example, consider an autosomal variant in which father is a heterozygous carrier, but mother and child are homozygous. If both mother and father are unaffected with the disease, then that particular variant is unremarkable and can likely be excluded from the analysis. However, if mother and child are both affected, then that particular variant would become interesting as a potential autosomal recessive variant. Using custom scripts to annotate variants based on these preconditions helps the genetic counselor or investigator balance the complexities of the analysis. This allows them to filter directly for autosomal variants rather than the different combinations of genotypes that correspond to the all of the possible autosome/sex-chromosome, affected status of the parents and sex of the child combinations. Another custom annotation that is commonly used is that for compound heterozygotes (CompoundHet), variants located in different places of the same gene in the affected child that are inherited from the mother (one variant) and from the father (the other variant). If phase information is available, the status of compound heterozygotes can be used to confirm that one variant was contributed by each parent, rather than the usual way of identifying genes with more than one mutation and assuming one is from each parent. Using these annotations, we can quickly remove variants that are unlikely to be the causal. In our NA12878 trio example (provided on the Web site), filtering based on InheritancePattern annotation (AR, deNovo, NonMendelian and XLD) eliminates variants that do not match any interesting inheritance mode. It decreases the total number of variants from 74 362 to 3008. Restricting variants to those with a predicted loss of function (SAVANT_IMPACT=HIGH) reduces this number to 21. Because of the exploratory nature of VCF-Miner, if the users need to apply a different filter such as the CompoundHet, they can save their analysis, clear the filters and then select CompoundHet=true. In this case, the number of variants drops to 11. The users can go back to their original analysis at any time. Clearly, these annotations would not always be available (or necessary) as they are specific to the experimental design; yet, they markedly decrease the number of variants suspected to be causative, thereby decreasing assessment time for that trio, which demonstrates the need for flexibility when working with diverse data sets and their respective annotations.

The other use case is for studies that have large numbers of samples that need to be analyzed in multiple groups. Using VCF-Miner with the 1KG.chr22.anno.vcf.bgz example file, there are three groups that we want to analyze. In Group 1, we have the first 38 samples (HG00098–HG00178), and Group 2, the next 7 samples (HG00179–HG00186). Suppose Group 1 and Group 2 show a phenotype of interest that is not present in Group 3, where Group 2 shows strong expression of the phenotype, Group 1 are moderate expressors and Group 3 does not express the phenotype. If we restrict the data to those that are homozygous in 50% of Group 2 and heterozygous in 50% of Group 1, and not in Group 3, and then restrict to SAVANT_IMPACT=HIGH, we quickly go from 348 110 variants to 84. This level of multi-grouping flexibility highlights the need for dynamic query applications.

## Discussion

VCF-Miner is the first publicly available GUI that allows non-bioinformatics experts to query the content of VCF files. The application can process any VCF as long as it is properly formatted. Compared with other GUI-based applications, VCF-Miner does not constrain the user to use a specific set of annotations. The flexibility is critical when considering the growing number of available sources of annotations. For instance, the 1000 Genomes Project recently released a new corpus of genetic variation found in their cohort. Allele frequency data for more than 81 million variants (www.1000genomes.org) is now available that can be used as filtering criteria by VCF-Miner. This data set can be downloaded and added to existing VCF files with relative ease, using annotation frameworks such as BioR [3] and used as filtering criteria by VCF-Miner.

VCF-Miner is a flexible tool that supports several filtering strategies, including the identification of somatic variants by comparing normal and tumor or identification of variants associated with rare diseases that involves the comparison between trios. Multiple groups allow queries to be resolved in experimental designs more complex than a standard case control.

Tools like vcf-validator [5] or ValidateVariants [2] can be used to ensure that data integrity is maintained—a principle not feasible with tab-delimited or Microsoft Excel files. Another advantage of VCF-Miner resides in its speed. Large data sets can be interactively filtered even when a large number of annotations have to be managed. McCarthy *et al*. [19] stressed the need to leverage multiple sources of annotations to truly understand the impact of variants because different annotation tools like ANNOVAR [4], SnpEFF [20] and Variant Effect Predictor [21] may yield different interpretations of the same variant.

Owing to its user-friendly interface, VCF-Miner is a tool that can be used by both bioinformaticians and non-computer programmers. The ability to export and reuse filtering strategies can help streamlining the filtering process and improves reproducibility. Future enhancements of VCF-Miner will allow exporting of VCFs, merge multiple VCFs and add annotations.

**Key Points**

- Use your own annotations to sort, query, and filter.
- Handles non-diploid genotypes.
- Fast filtering of millions of variants.
- Add and query based on any number of user-defined groups.
- Save and reuse analysis plans for reproducible research.

## Funding

This work was supported by the Center for Individualized Medicine at Mayo Clinic.
